# Denoising method of machine tool vibration signal based on variational mode decomposition and Whale-Tabu optimization algorithm

**DOI:** 10.1038/s41598-023-28404-7

**Published:** 2023-01-27

**Authors:** Chengzhi Fang, Yushen Chen, Xiaolei Deng, Xiaoliang Lin, Yue Han, Junjian Zheng

**Affiliations:** 1 Key Laboratory of Air-Driven Equipment Technology of Zhejiang Province, Quzhou University, Quzhou, 324000 China; 2 College of Mechanical Engineering, Zhejiang University of Technology, Hangzhou, 310023 China

**Keywords:** Engineering, Mathematics and computing

## Abstract

The noise from other sources is inevitably mixed in the vibration information of CNC machine tools obtained using the sensors. In this work, a de-noising method based on joint analysis is proposed. The variational mode decomposition (VMD), correlation analysis (CA), and wavelet threshold (WT) denoising are used to denoise the original signal. First, VMD decomposes noisy signals into multiple intrinsic mode functions (IMFs). The penalty factor and decomposition level of VMD parameters are selected by the optimization algorithm by combining the whale optimization algorithm (WOA) and tabu search (TS). The minimum permutation entropy of IMF is used as the fitness function of the proposed fusion algorithm. Then, the IMF is divided into three categories by using the cross-correlation number. They include the pure components, signals containing noise, and complete noise components. Then, the WT method is used to further denoise the signals, and signal reconstruction is performed with the pure component to obtain the denoised signal. This joint analysis denoising method is named TS-WOA-VMD-CA-WT. The simulation results show that the fusion optimization algorithm proposed in this work has better performance as compared to the single optimization algorithm. It performs effectively when applied to the actual machine tool vibration signal denoising. Therefore, the proposed TS-WOA-VMD-CA-WT method is superior to other existing denoising techniques and has good generality, which is expected to be popularized and applied more widely.

## Introduction

Being a critical component in manufacturing industry, it is important to improve the machining accuracy of CNC machines^1^. The vibrations caused due to machine operations and external environment are an key factor affecting the machining accuracy of CNC machine tools. With the development of intelligent manufacturing, the physical information can be transformed into the digital information based on various sensors and devices. As a result, intelligent sensing can be applied for performing analysis and decision-making, and finally for devising intelligent product design, and realizing manufacturing and production^2^. Monitoring the condition of machining tools based on sensors can provide additional information related to the the state of the machine tool^3^. However, when acquiring machine tool vibration information, human-related factors, environment, and the influence of the sensors inevitably lead to the addition of noise in the original signals. The direct use of these signals is not conducive for further analysis. Therefore, it is necessary to de-noise the collected vibration signals of machine tools so that they can be effectively used in a larger field.

There are various works presented in literature that focus on signal denoising. Fourier transform^4^ is a well-known signal analysis technique. However, this method can not be used for local analysis of non-stationary signals, and the effect of noise reduction is poor. As compared with the Fourier transform, the wavelet transform has various advantages, including nonlinearity, locality, and good time–frequency characteristics^5^, thus making it very suitable for analyzing the abrupt and non-stationary signals. Wang et al.^6^ preprocessed the bridge vibration signals based on WT denoising to effectively reduce the interference of random noise and obtain accurate natural vibration characteristics of bridge structures. Chen et al.^7^ proposed a correlation calculation method based on wavelet packet transform for denoising the blasting vibration signals, which retained the real characteristics of blasting vibration signals. This method is also used in fault diagnosis^8^, health monitoring^9^, and electronic communication, etc.

The empirical mode decomposition (EMD)^10^ effectively addresses the limitation of time–frequency analysis method and is no longer constrained by linearity and stationarity. It is based on the concept of IMF, which adaptively generates the basis functions, i.e., the IMFs generated by the screening process is its basis function. This method is suitable for the analysis of nonlinear and non-stationary signals, and achieves high accuracy in time and frequency domains. Although EMD is an adaptive time–frequency analysis method, its end effect and mode aliasing hinder the development and application of EMD. As improved algorithms for EMD, such as ensemble empirical mode decomposition (EEMD)^11^, which takes advantage of the uniform frequency distribution of white Gaussian noise and performs empirical mode decomposition several times by adding white Gaussian noise. Jia et al.^12^ proposed a vibration signal denoising method based on EEMD and grey theory to effectively extract the vibration feature information of hob tools. Zheng et al.^13^ also proposed a mean optimization decomposition method to improve the performance of EMD in mean curve construction.

The variational mode decomposition (VMD) is an adaptive and completely non-recursive signal processing algorithm^14^. As compared with EMD and the improved EMD algorithm, VMD not only has a solid theoretical basis, but is robust to sampling and noise^15^. Xiao et al.^16^ proposed an adaptive denoising algorithm based on probability density function and VMD, and they evaluated the effectiveness of the method through mean square error and signal-to-noise ratio. The result of VMD is determined by the penalty factor *α* and the number of decomposition layers *k*. The adaptive parameter optimization VMD method proposed in^17^ determines the optimal number of decomposition layers *k* by analyzing the ratio of the center frequencies of two adjacent IMFs. Yu et al.^18^ determined the number of decomposition layers of VMD by using permutation entropy and verified the superiority of this method based on the simulated and actual vibration signals. Hu et al.^19^ constructed an improved optimization algorithm, and computed the root mean square error as the fitness function to obtain the number of decomposition layers and the optimal penalty factor.

In order to reduce the noise from the vibration signals of machine tools, this work proposes a joint analysis denoising method, which consists of VMD, correlation analysis, and WT denoising. The preset parameters *α* and *k* in VMD are obtained by the parameter optimization algorithm fused with whale optimization algorithm (WOA) and tabu search (TS). This name of this method is TS-WOA-VMD-CA-WT. The minimum permutation entropy of decomposed IMFs are computed as the fitness function, and [*α*, *k*] is considered as the population individual. The optimal fitness value is searched based on the fusion algorithm to decompose.Then, according to the correlation coefficient, the decomposed imf is divided into the following categories, including complete noise components, signals containing noise, and pure components. The complete noise components are discarded and the pure components are retained. Then, the signals containing noise are processed by using WT and the signal is reconstructed to obtain the denoised signal.

The rest of this manuscript is organized as follows. Section “Signal denoising theory” presents the basic theory of WT and VMD methods. Section “Intelligent Optimization Algorithm” presents the basic theory of WOA and TS. Section “Method” presents the fusion algorithm and the overall process of noise reduction. Section “Simulations” verifies the effectiveness of TS-WOA-VMD-CA-WT based on two groups of analog signals. Section “Application of machine tool vibration test” applies this method to the vibration signal processing of test machine tools to denoise the real-world data. Finally, Section “conclusion” concludes this work.

## Signal denoising theory

### Variational mode decomposition (VMD)

VMD is an adaptive signal decomposition method, and the major steps of its decomposition are summarized below^17^:

The signal is decomposed adaptively into *k* modal functions with central frequency $\omega_{k}$, i.e., the intrinsic modal function. The *k*-th order IMF component is mathematically expressed as:

1 $$ \mu_{k} (t) = A_{k} (t)\cos (\varphi_{k} (t)) $$

where, $A_{k} (t)$ and $\varphi_{k} (t)$ are the instantaneous amplitude and frequency of $\mu_{k} (t)$ respectively .

Apply Hilbert transform on each mode function $\mu_{k} (t)$ and calculate the corresponding analytical signal The exponential harmonic signal $e^{{j\varphi_{k} (t)}}$ is added in each modal component to correct the central frequency of the analytical signal of each mode. Then, each modal component is modulated to the corresponding fundamental frequency band. In order to estimate the corresponding bandwidth of each modal component, a constrained variational model is constructed as follows.

2 $$ \left\{ \begin{gathered} \mathop {\min }\limits_{{\{ \mu_{k} \} ,\{ \varphi_{k} \} }} \left\{ {\sum\limits_{k = 1}^{K} {\left\| {\partial_{t} \left[ {(\delta (t) + \frac{j}{\pi t})*\mu_{k} (t)} \right]*e^{{ - j\varphi_{k} (t)}} } \right\|_{2}^{2} } } \right\} \hfill \\ s.t.\sum\limits_{k = 1}^{K} {\mu_{k} (t) = x(t)} \hfill \\ \end{gathered} \right. $$

where,$\delta (t)$ denotes the unit impulse function, *j* denotes the imaginary unit, and *t* denotes the time. $\left\{ {u_{k} } \right\}$ and $\left\{ {\omega_{k} } \right\}$ denote the set of modal components and the corresponding center frequencies, respectively, $\delta (t)$ denotes the Dirichlet function, and $\partial_{t}$ represents the derivative with respect to time. To obtain the optimal solution of (2), the Lagrange multiplier $\lambda$ and the quadratic penalty factor $\alpha$ are introduced as follows.

3 $$ \begin{gathered} L(\left\{ {u_{k} } \right\},\left\{ {\omega_{k} } \right\},\lambda ) = \hfill \\ \alpha \sum\limits_{k = 1}^{K} {\left\| {\partial_{t} \left[ {(\delta (t) + \frac{j}{\pi t})*\mu_{k} (t)} \right]*e^{{ - j\varphi_{k} (t)}} } \right\|_{2}^{2} } \hfill \\ + \left\| {f(t) - \sum\limits_{k = 1}^{K} {u_{k} (t)} } \right\|_{2}^{2} + \lambda (t),f(t) - \sum\limits_{k = 1}^{K} {u_{k} (t)} \hfill \\ \end{gathered} $$

The alternating direction method of multipliers (ADMM) is used to iterate multiple sub-optimization problems. $u_{k}^{n + 1}$, $\omega_{k}^{n + 1}$ and $\lambda^{n + 1}$ are alternately updated to find the optimal solution of the above problem^20^.

### Correlation analysis

Correlation analysis usually uses correlation coefficient, and it can measure the independence and correlation between the data points^21^, and is mathematically defined as follows:

4 $$ R = \frac{{E\left( {u_{k} (t)x(t)} \right) - E\left( {u_{k} (t)} \right)E\left( {x(t)} \right))}}{{\sqrt {D\left( {u_{k} (t)} \right)} \sqrt {D\left( {x(t)} \right)} }} $$

where, $x(t)$ and $u_{k} (t)$ represent the original signal and the *k*th IMF component obtained using VMD, respectively. The closer the absolute value of R is to 1, the higher is the correlation between $x(t)$ and $u_{k} (t)$.

### Wavelet threshold denoising (WT)

In this work, we adopt the soft threshold method for denoising, and the original signal *x*(*t*) is decomposed based on *n* layers of wavelet. The low frequency coefficient $\alpha_{i}$ and high frequency coefficient $d_{i}$ from each layer are extracted. Then, the threshold ${\text{thr}} = \sigma \sqrt {2\log N}$ is determined, where $\sigma$ represents the standard deviation of noise and *N* denotes the length of the signal. The high frequency coefficient is processed by the soft threshold function as follows[26]:

5 $$ x_{i}^{^{\prime}} (t) = \left\{ \begin{gathered} {\text{sgn}} \left( {x_{i} (t)} \right)\left( {\left| {x_{i} (t) - {\text{thr}} } \right|} \right) \, \left| {x_{i} (t)} \right| \ge {\text{thr}} \hfill \\ 0 \, \left| {x_{i} (t)} \right| < {\text{thr}} \, \hfill \\ \end{gathered} \right. $$

where, $x_{i} (t)$ denotes the high-frequency wavelet coefficient of layer *i* in wavelet decomposition and $x_{i}^{^{\prime}} (t)$ denotes the high-frequency wavelet coefficient of layer *i* after threshold processing.

## Intelligent optimization algorithm

### Whale optimization algorithm (WOA)

WOA is a meta-heuristic optimization algorithm developed to imitate whale predation behavior and it has simple mechanism and strong universality. Suppose that each ***X*** is an individual whale among the population participating in the hunt, and *p* denotes the probability of predation strategy, which satisfies the random distribution of [0, 1]. This process comprises following major steps^22^:

#### Step 1: Surround the prey.

When the probability *p* < 0.5 and the coefficient vector $\left| {\varvec{A}} \right| \le 1$, the whales identify the prey and surround it. The current location of the prey is marked as ${\varvec{X}}_{t}^{*}$. Other whales update their position by swimming towards ${\varvec{X}}_{t}^{*}$. During this process, the positions of the whales are updated as follows:

6 $$ {\varvec{X}}_{t + 1} = {\varvec{X}}_{t}^{*} - {\varvec{A}} \cdot \left| {{\varvec{C}} \cdot {\varvec{X}}_{t}^{*} - {\varvec{X}}_{t} } \right| $$

where, ${\varvec{X}}_{t + 1}$ denotes the updated position vector, ${\varvec{X}}_{t}$ denotes the position vector, ${\varvec{X}}_{t}^{*}$ denotes the current optimal solution, and ***A*** and ***C*** represent the coefficient vectors.

#### Step 2: Spiral bubble attack.

When the coefficient vector $\left| {\varvec{A}} \right| \le 1$ and the probability of the whale's predation strategy $p \ge 0.5$, the whales use bubbles to attack the prey and move toward the optimal whale in a spiral manner. In this process, the position of the whale is updated as follows:

7 $$ {\varvec{X}}_{t + 1} = {\varvec{X}}_{t}^{*} + {\varvec{D}} \cdot e^{bl} \cdot \cos (2\pi l) $$

where, D denotes the search distance that satisfies ${\varvec{D}} = \left| {{\varvec{C}} \cdot {\varvec{X}}_{t}^{*} - {\varvec{X}}_{t} } \right|$. *b* denotes the helical shape constant, which is usually equal to 1. *l* denotes a random number ranging from [− 1, 1] and its size determines the distance of an individual whale from the optimal whale.

#### Step 3: Random search for prey.

When the constriction and encircling mechanism is not satisfied at $\left| {\varvec{A}} \right| > 1$, the whales no longer move towards the location of current optimal whale. Instead, they move randomly. During this process, the position of the whale is updated as follows:

8 $$ {\varvec{X}}_{t + 1} = {\varvec{X}}_{t}^{r} - {\varvec{A}} \cdot \left| {{\varvec{C}} \cdot {\varvec{X}}_{t}^{r} - {\varvec{X}}_{t} } \right| $$

where, ${\varvec{X}}_{t}^{r}$ denotes the position vector of a whale randomly selected from the current population.

### Tabu search algorithm (TS)

The TS algorithm is based on the initial solution. This algorithm uses tabu and amnesty criteria, thus enabling it to avoid the repeated search during the search process. In addition, it can effectively jump out of the local optimal solution, and finally obtains the global optimal solution. The major steps of this process are presented below: 1. Set the relevant parameters of tabu algorithm. 2. Generate a neighborhood based on the current solution and select an appropriate solution in the neighborhood as the candidate solution. 3. Conduct corresponding operations according to whether the candidate solution meets the contempt criterion^23^. 4. Repeat steps 2–3 until the maximum number of iterations is reached.

## Method

In this work, the advantages of two aforementioned methods are combined to construct a new optimization algorithm, i.e., TS-WOA-VMD, which assists the whale algorithm to avoid the local optima with the support of tabu table. At the same time, the key to the optimization algorithm is the selection of fitness function. This work adopts the minimum permutation entropy as the fitness function. As a parameter used for measuring the complexity of chaotic time series, the permutation entropy has stronger anti-interference ability, and better robustness^24^. The smaller the entropy value is, the more regular is the time series distribution, thus showing that the IMF contains more effective information. Based on the theory presented in Sections “Signal denoising theory” and “Intelligent Optimization Algorithm”, this work proposes a joint analysis noise reduction method, named TS-WOA-VMD-CA-WT. The major steps of the proposed algorithm are presented below:

### Step 1:

The parameters of the signal to be denoised are optimized based on the TS-WOA-VMD optimization algorithm.

1. The tabu table and the parameters required by the algorithm are initialized. The quadratic penalty term α in VMD and the number of decomposition levels k are defined as population individuals (*α*, *k*). The tabu objects are set as the lower limits of two variables *lb*_1_ and *lb*_2_ in the WOA in order to control the search area of the whales and conduct accurate local optimization.
2. After setting the first tabu object, whale algorithm is initialized for computing the initial solution *L* = 1.
3. In the WOA-VMD program, the VMD is performed for the first-generation population. We set G = 1 and calculate the minimum permutation entropy value of k IMF components. This computed value is recorded as the initial value in the iteration curve. The position update method is judged according to the value of $\left| {\varvec{A}} \right|$, the update method referring to (6), (7), and (8). Then, the next cycle (G = G + 1) is performed. The WOA is executed until the maximum number of iterations is reached, or the convergent whale optimal position, namely the local optimal point, is obtained.
4. The local optimum also represents the solution of TS. After the initial solution is determined, it is tentatively determined as the global optimal solution, and the candidate solution is generated by neighborhood search and solved by using 3). If the candidate solution is better than the current global optimal solution, it is replaced. On the other hand, if it is not better than the current global optimal solution, the optimal solution that is not tabu in the candidate solution is replaced, so as to update the current solution in the tabu table. Then, the next iteration (*L* = *L* + 1) is started. Repeat this process until reach the maximum number of iterations, and then stop. Based on the tabu object corresponding to the global optimal solution, i.e., the parameters *α* and *k*, finally VMD is performed.

### Step 2:

After VMD processing based on optimized parameters, *k* IMF components are obtained. According to^15^, the threshold of correlation coefficient is set to 0.2. The IMF whose correlation coefficient is less than the threshold is regarded as noise components. The largest IMF component of correlation coefficient is the pure component. The rest are components containing noise.

### Step 3:

Retain the pure component and discard the complete noise component. For the signals containing noise, the wavelet three-layer decomposition and soft threshold function are used for denoising.

### Step 4:

**Figure 1:**
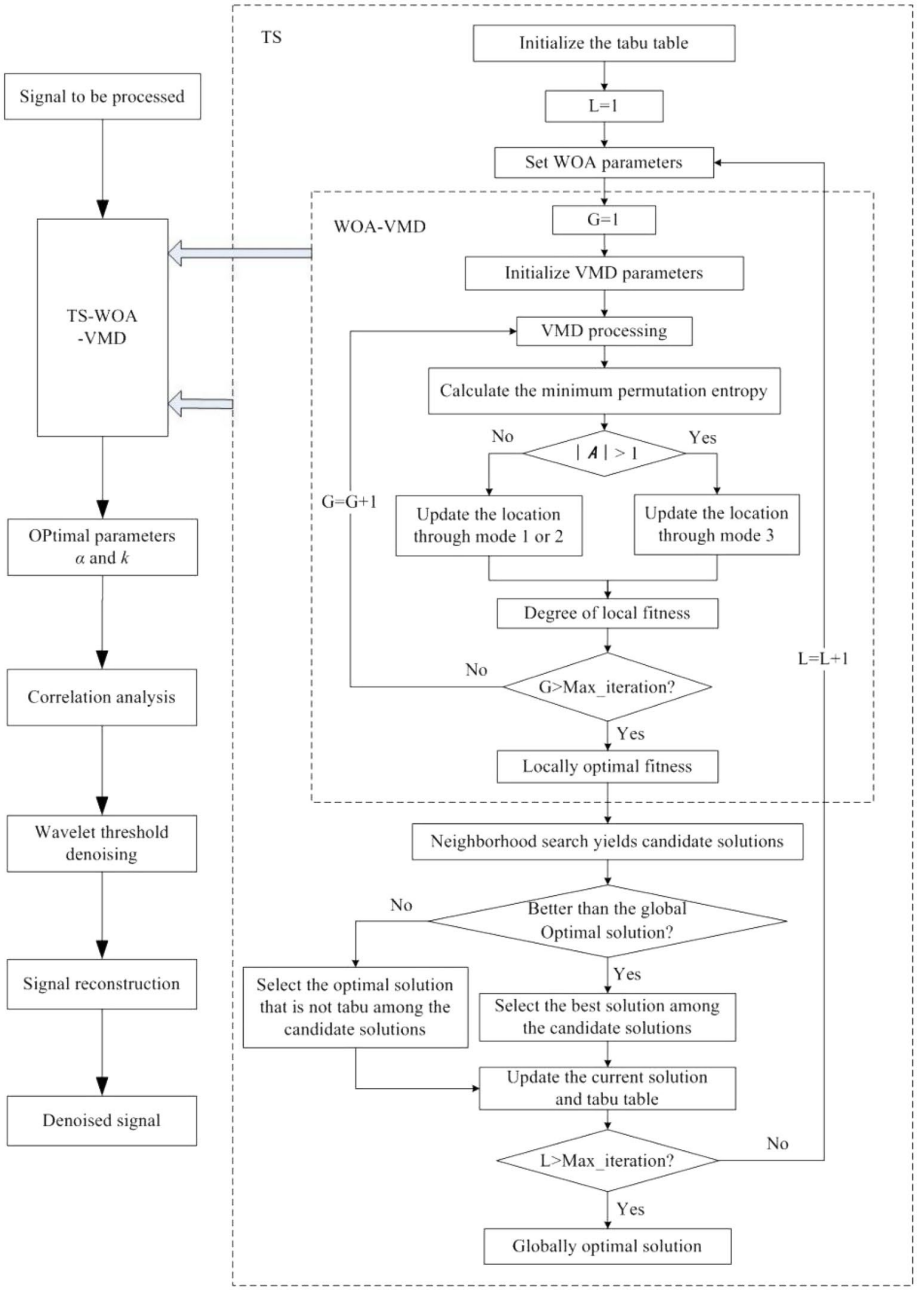
The denoising process based on the proposed TS-WOA-VMD-CA-WT.

Reconstruct the useful signal components to obtain the denoised signal. The flow of TS-WOA-VMD-CA-WT algorithm proposed in this work for signal denoising is shown in Fig. 1.

## Simulations

To verify the superiority of the proposed method for denoising, this work first performs denoising experiments on the simulation signals. The machine tool is affected by various environmental factors, and the noise source and intensity are constantly changing. In this section, two analog signals, i.e., single frequency and noise signal, and mix of frequency and noise signals, are sampled at 1000 Hz for the construction of simulation signals. The two signals are defined as follows:

9 $$ c_{1} (t) = A_{1} \sin (2\pi f_{1} t) $$

10 $$ \begin{gathered} c_{2} (t) = A_{2} \sin (2\pi f_{2} t) + A_{3} \sin (2\pi f_{3} t) \hfill \\ \, + A_{4} \sin (2\pi f_{4} t) \hfill \\ \end{gathered} $$

where, *f*_1_ = 50 and *A*_1_ = 2 represent the amplitude and frequency of *c*_1_ signal, respectively. *f*_2_ = 40, *f*_3_ = 50, *f*_4_ = 10, *A*_2_ = 1, *A*_3_ = 2, and *A*_4_ = 3 represent the three different frequencies of *c*_2_ mixed frequency signal and their corresponding amplitudes, respectively. Afterwards, the white Gaussian noise with different intensity is added respectively in the signal to simulate the actual situation. This is mathematically expressed as follows:

11 $$ x_{ij} (t) = c_{i} (t) + n_{j} (t) $$

where, $c_{i} (t)$(*i* = 1, 2) represents the pure signal, $n_{j} (t)$(*i* = 1, 2, 3, 4, 5) represents the white Gaussian noise. Please note that random noise signals of 10 dB, 5 dB, 0 dB, -5 dB, and -10 dB are added for simulating the signals affected by different intensities of noise. $x_{ij} (t)$ denotes the signal to be denoised. $x_{ij} (t)$ is the signal processed by the proposed joint denoising analysis.

method TS-WOA-VMD-CA-WT. The denoising effect is evaluated by using *SNR* and mean square error *RMSE*, which are mathematically expressed as follows:

12 $$ SNR = 10\lg \left( {\frac{{\sum\limits_{m = 1}^{M} {x_{i}^{2} } }}{{\sum\limits_{m = 1}^{M} {(x_{i} - x\prime_{i} )^{2} } }}} \right) $$

13 $$ RMSE = \sqrt {\frac{1}{m}\sum\limits_{i = 1}^{m} {\left( {x^{\prime}_{i} - c_{i} } \right)^{2} } } $$

where, m represents the length of the signal, *x*_*i*_ represents the original signal sequence, $x^{\prime}_{i}$ represents the denoised signal sequence, and *c*_*i*_ is the simulation signal sequence. The larger SNR and smaller RMSE indicate a better signal denoising effect.

The proposed method is compared with other denoising methods, including WOA-VMD-CA-WT, TS-VMD-CA-WT, EMD-CA-WT and EEMD-CA-WT. In the two former algorithms, the proposed fusion algorithm is replaced by a single optimization algorithm, and in the two later algorithms, the VMD decomposition is replaced by the EMD and EEMD methods, respectively.

### Single frequency signal denoising

**Figure 2:**
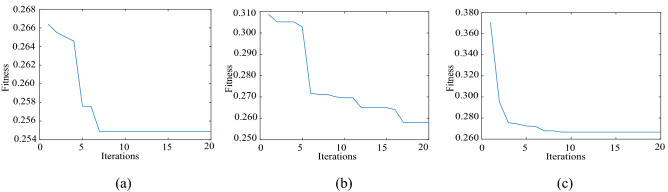
The iterative process of the proposed algorithm under 5 dB. (**a**) TS-WOA-VMD. (**b**) WOA-VMD. (**c**) TS-VMD.

**Figure 3:**
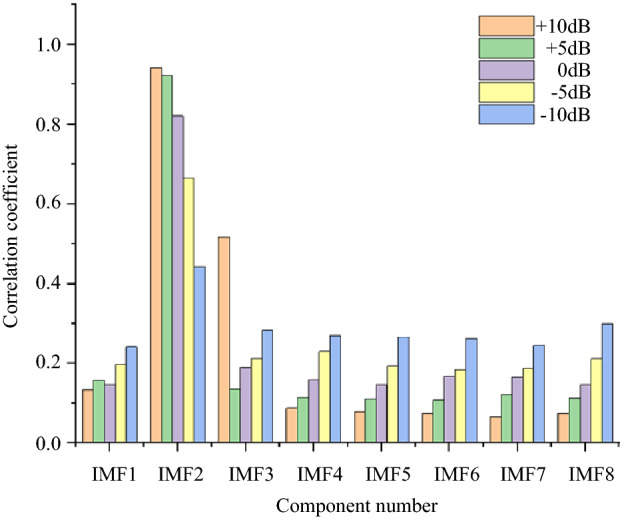
Correlation coefficients corresponding to different IMFs.

**Figure 4:**
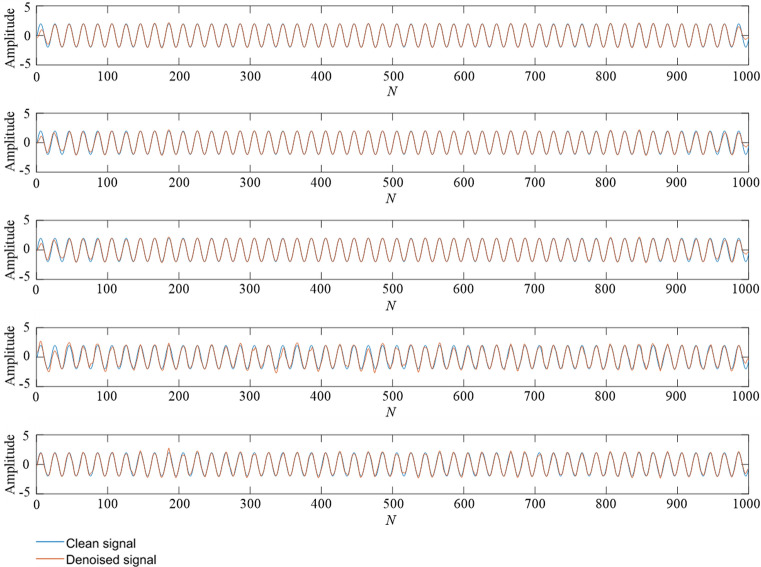
A comparison of denoising effect comparison under 10 dB. From top to bottom: (**1**) TS-WOA-VMD-CA-WT; (**2**) WOA-VMD-CA-WT; (**3**) TS-VMD-CA-WT; (**4**) EMD-CA-WT; (**5**) EEMD-CA-WT.

**Figure 5:**
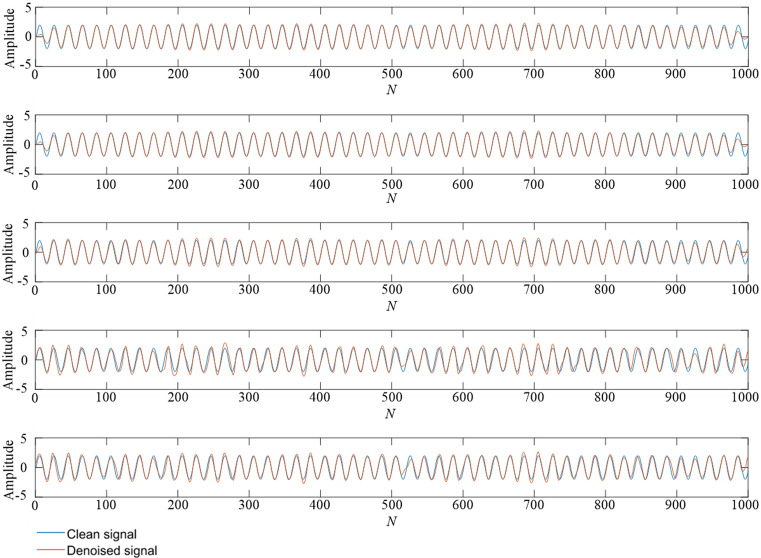
A comparison of denoising effect under 0 dB. From top to bottom: (**1**) TS-WOA-VMD-CA-WT; (**2**) WOA-VMD-CA-WT; (**3**) TS-VMD-CA-WT; (**4**) EMD-CA-WT; (**5**) EEMD-CA-WT.

**Figure 6:**
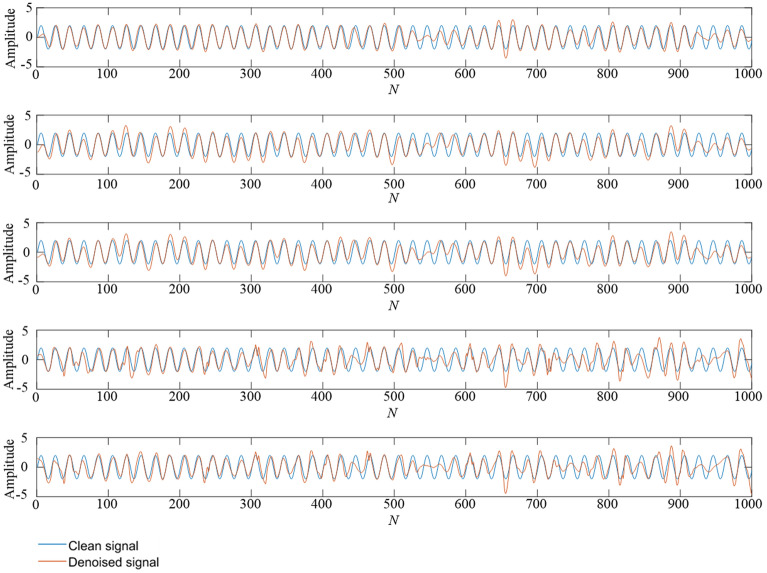
A comparison of denoising effect under -10 dB. From top to bottom: (**1**) TS-WOA-VMD-CA-WT; (**2**) WOA-VMD-CA-WT; (**3**) TS-VMD-CA-WT; (**4**) EMD-CA-WT; (**5**) EEMD-CA-WT.

For the simulation signal $c_{1} (t)$, five noise signals with different intensities are added to obtain five groups of original signals. The method described in Section “Method” is used to denoise the five groups of signals. Finally, we will take the simulation signal containing 10 dB, 0 dB, − 10 dB noise to show the denoising effect. Table 1 shows the initialization parameters of the fusion optimization algorithm. Figure 2 shows the iterative processes of TS-WOA-VMD, WOA-VMD and TS-VMD algorithms for achieving optimization. Figure 3 shows the correlation coefficients of each modal component after the decomposition of the $c_{1} (t)$. Figures 4, 5, 6 show the signal denoising results for 10 dB, 0 dB, and -10 dB original signals by using TS-WOA-VMD-CA-WT, WOA-VMD-CA-WT, TS-VMD-CA-WT, EMD-CA-WT, and EEMD-CA-WT methods, respectively. Table 2 shows the denoising effects of five denoising methods on five groups of signals with different degrees of noise. The values in bold highlight the results based on the method presented in this paper.

**Table 1:** The initial parameters of the optimization algorithm.

Parameter	Region or value
Penalty factor: *α*	$\alpha \in [1000,\;20000],\;\alpha \in Z$
Number of IMFs: *k*	$k \in [3,\;12],\;k \in Z$
Step length of TS: *h1* & *h2*	1000 & 1
The span of the upper and lower limits: *d1* & *d2*	2000 & 3
The length of the neighborhood	5
Number of candidate solutions	5
Initial solution *lb*_1_ & *lb*_2_	5000 & 7
The size of population for WOA	20
The maximum iteration of TS & WOA	20

**Table 2:** The denoising effect of five methods for single frequency simulation signal.

Noise intensity	Indicator	EMD-CA-WT	EEMD-CA-WT	TS-VMD-CA-WT	WOA-VMD-CA-WT	TS-WOA-VMD-CA-WT
+ 10 dB	RMSE	0.3248	0.1881	0.1847	0.1849	**0.1571**
	SNR	12.8272	17.4676	17.3932	17.3854	**18.8987**
+ 5 dB	RMSE	0.3920	0.3441	0.2173	0.2151	**0.2143**
	SNR	11.2907	12.5846	16.2832	16.3069	**16.3386**
+ 0 dB	RMSE	0.5267	0.4338	0.2706	0.2603	**0.2600**
	SNR	9.2311	10.5910	14.5909	14.6792	**14.6841**
− 5 dB	RMSE	0.8428	0.7009	0.5413	0.3415	**0.3364**
	SNR	5.8859	7.0212	9.2685	12.6553	**12.8218**
− 10 dB	RMSE	1.0186	0.9306	0.8460	0.8134	**0.5818**
	SNR	3.1336	3.6410	4.7740	5.2666	**7.2527**

Figure 2 shows the iterative process of VMD parameter optimization of the three algorithms by considering the 5 dB original signal. The Figures show that different algorithms achieve convergence within 20 epochs. The minimum fitness values of the fusion algorithm, WOA, and TS proposed in this paper are 0.2554, 0.2580, and 0.2667, respectively. The smaller the permutation entropy is, the better the signal decomposition performance is. Therefore, the proposed fusion algorithm achieves the optimal effect and effectively improves the ability of parameter optimization. The whale algorithm is prone to fall into the local optima in the later stages due to its enveloping characteristic. As a result, the best VMD parameters cannot be obtained. Although the TS avoids the cycle of the search process based on the tabu table in the limited space, it is difficult to find the exact solution outside the range of step size. In the proposed method, because of the addition of tabu table, the whale method can perform accurate search in the local scope, while avoiding the repeated search in the global scope. As a result, it effectively avoids the problems, i.e., the whale algorithm is prone to falling in the local optima and the TS is unable to find the exact solution.

In Fig. 3, the first 8 signal components are selected for comparison due to the different optimal parameters obtained by different original signals and the number of decomposition layers. It can be found that as the signal-to-noise ratio decreases, the correlation coefficient tends to average gradually, which indicates that when the signal-to-noise ratio is small, the effective information in the obtained modal component will decrease. In this case, the useful information contained in IMF2 will flow to other components. As usual, for those signals which are highly disturbed by noise, it is necessary to extract effective information from other components by means of wavelet threshold for further reconstruction.

Figures 4, 5, 6 show that no matter which method is used, it can de-noise the mixed noise signal and improve its matching degree with the pure signal. The decibel value here represents the ratio between the effective part and the noise part of the signal. Therefore, the process of adding 10 dB, 5 dB, 0 dB, − 5 dB, and − 10 dB white Gaussian noise gradually increases the influence on the pure signal. It is evident from Table 2 that with an increase in the proportion of the original signal noise, the index of SNR decreases, while the value of RMSE keeps increasing. This indicates that the denoising effect of various denoising methods decreases to a certain extent. Based on the horizontal analysis, i.e., the comparison of the same signal decibels, the TS-WOA-VMD-CA-WT method proposed in this work achieves the optimal effect for five kinds of noisy signals. The proposed method has the smallest mean square error and largest SNR. WOA-VMD-CA-WT and TS-VMD-CA-WT are second in terms of performance, whereas EEMD-CA-WT and EMD-CA-WT have low performance. The denoising performance of EEMD is better than EMD as EEMD effectively.

In addition, as compared with the decomposition methods of EMD and EEMD, the denoising result of VMD is smoother and closer to original signal without sharp and burr phenomenon. This is because the VMD overcomes the mode aliasing problem in EMD and determines the number of mode decomposition. For different signals, VMD can adaptively adjust its decomposition components to achieve the optimal effect. EEMD is essentially an improved EMD and the white noise is added in the decomposition process to avoid the appearance of mode aliasing. However, due to its characteristics, it inevitably introduces residual noise, which also affects its denoising performance. The method of TS-WOA-VMD-CA-WT obtains the optimal penalty factor and decomposition level of VMD by using fusion algorithm, thus making the denoising results closer to the pure signal. Moreover, it effectively avoids the burr and signal distortion.

### Mixed frequency signal denoising

**Figure 7:**
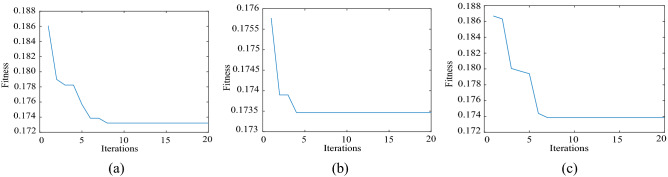
The iterative process of the proposed algorithm under 5 dB. (**a**) TS-WOA-VMD. (**b**) WOA-VMD. (**c**) TS-VMD.

**Figure 8:**
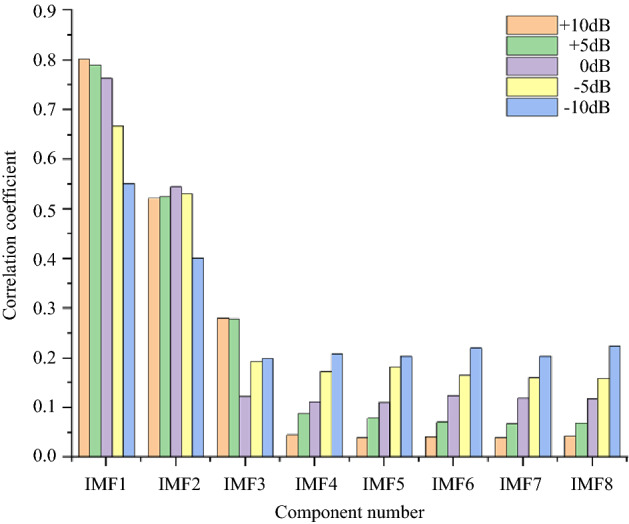
Correlation coefficients corresponding to different IMFs.

**Figure 9:**
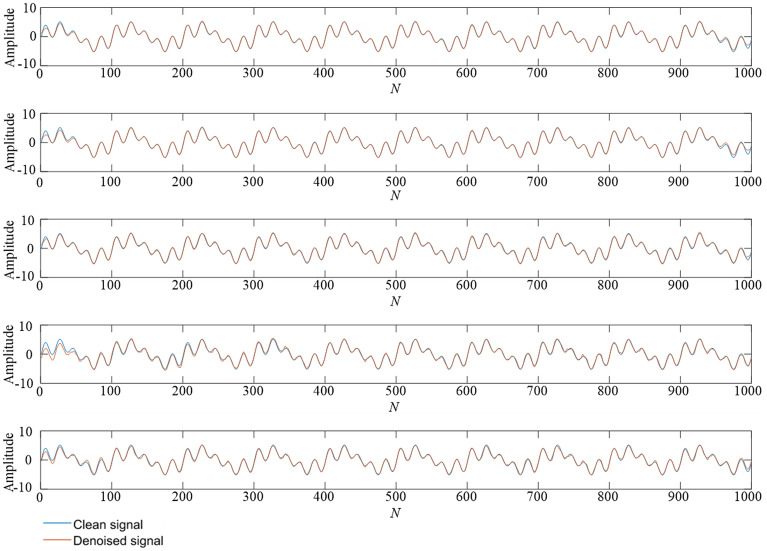
A comparison of denoising effect under 10 dB. From top to bottom: (**1**) TS-WOA-VMD-CA-WT; (**2**) WOA-VMD-CA-WT; (**3**) TS-VMD-CA-WT; (**4**) EMD-CA-WT; (**5**) EEMD-CA-WT.

**Figure 10:**
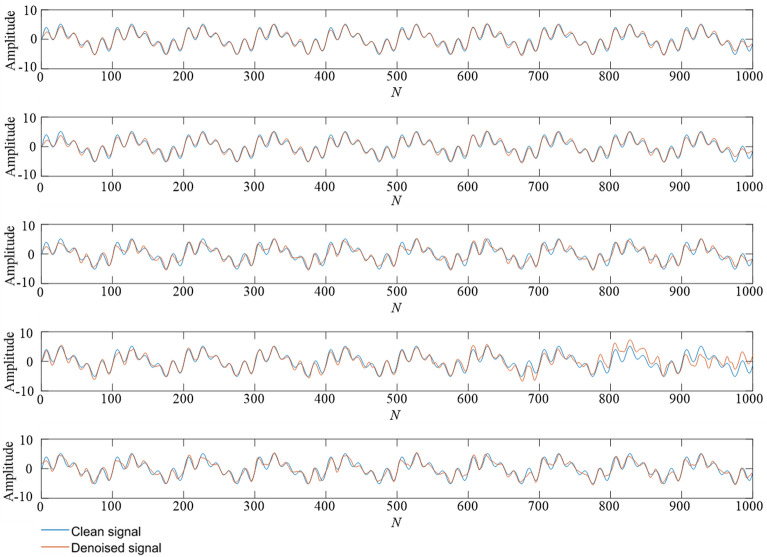
A comparison of denoising effect under 0 dB. From top to bottom: (**1**) TS-WOA-VMD-CA-WT; (**2**) WOA-VMD-CA-WT; (**3**) TS-VMD-CA-WT; (**4**) EMD-CA-WT; (**5**) EEMD-CA-WT.

**Figure 11:**
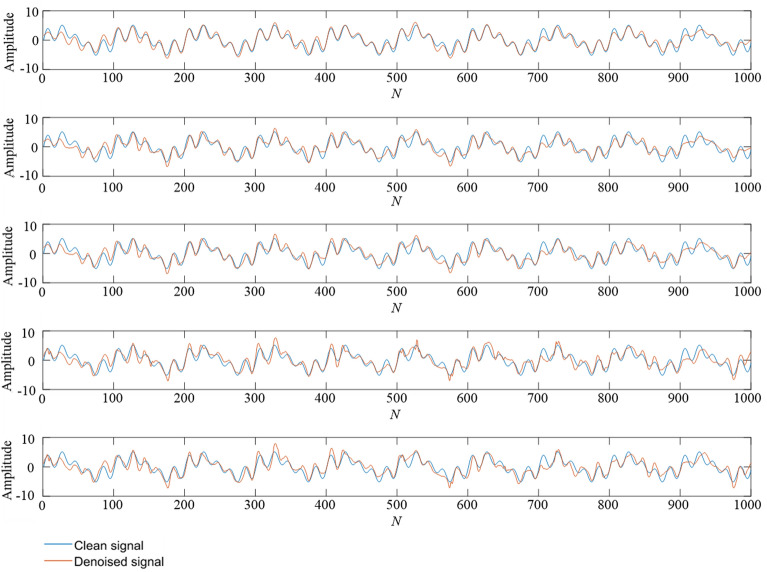
A comparison of denoising effect under -10 dB. From top to bottom: (**1**) TS-WOA-VMD-CA-WT; (**2**) WOA-VMD-CA-WT; (**3**) TS-VMD-CA-WT; (**4**) EMD-CA-WT; (**5**) EEMD-CA-WT.

When dealing with mixed frequency signals, the white Gaussian noise of 10 dB, 5 dB, 0 dB, − 5 dB, and − 10 dB is added in the original analog signal. The parameters of the fusion algorithm are initialized as presented in Table 1. The denoising process based on TS-WOA-VMD-CA-WT, WOA-VMD-CA-WT, TS-VMD-CA-WT, EMD-CA-WT, and EEMD-CA-WT methods is performed. The iterative results of the parameter optimization algorithm are shown in Fig. 7. The correlation coefficients of each modal component of the signal are shown in Fig. 8. The results of five signal denoising methods are presented in Table 3.The values in bold highlight the results based on the method presented in this paper. Figures 9, 10, 11 shows the effect drawings of mixed frequency signal denoising with 10 dB, 0 dB and − 10 dB noise respectively.

**Table 3:** The denoising effect of five methods for mixed frequency simulation signals.

Noise intensity	Indicator	EMD-CA-WT	EEMD-CA-WT	TS-VMD-CA-WT	WOA-VMD-CA-WT	TS-WOA-VMD-CA-WT
10db	RMSE	0.4297	0.3153	0.2250	0.2336	**0.1906**
	SNR	15.7530	17.8848	21.3406	20.9175	**22.7397**
5db	RMSE	0.9150	0.6665	0.6730	0.5020	**0.2751**
	SNR	9.0169	11.7289	11.5550	14.0098	**19.6446**
0db	RMSE	1.1660	0.7305	0.7608	0.5704	**0.4647**
	SNR	7.3636	10.8547	10.3003	12.8753	**14.8075**
− 5db	RMSE	0.9630	0.8981	0.8503	0.6397	**0.5952**
	SNR	8.7674	9.3238	9.5828	11.9271	**12.7117**
− 10db	RMSE	1.3259	1.3159	1.1091	1.1011	**0.9299**
	SNR	6.2013	6.2645	7.2902	7.0898	**8.8481**

In Fig. 7, the minimum fitness values obtained by three different parameter optimization algorithms are 0.1732, 0.1745, and 0.1739, respectively. It is evident that for the simulation signals with mixed frequencies, the fusion algorithm proposed in this work still achieves better results during the optimization process, which is helpful for subsequent denoising. In Figs. 9, 10, 11, the curve obtained after denoising by using TS-WOA-VMD-CA-WT method is more consistent with the pure signal and is smoother as well. Meanwhile, the analysis presented in Table 3 shows that the proposed algorithm in mixed frequency signal denoising effectively removes the noise components. For the proposed method, all signal-to-noise ratios reach the maximum and all root mean square errors reach the minimum, thus showing the versatility and stability.

**Figure 12:**
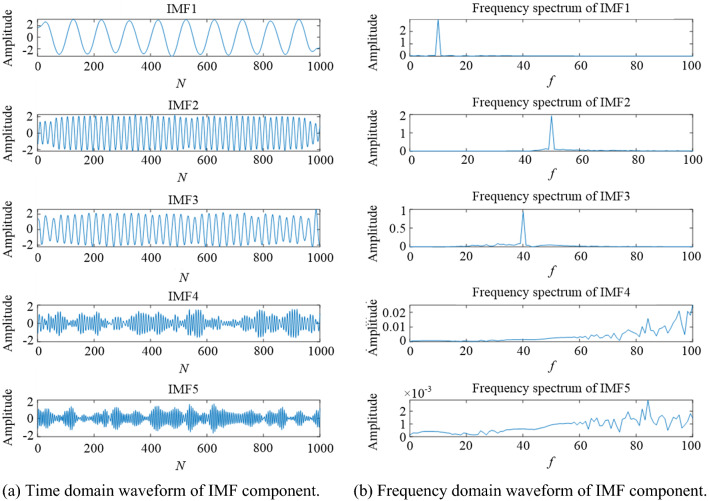
The component waveforms after VMD processing.

**Figure 13:**
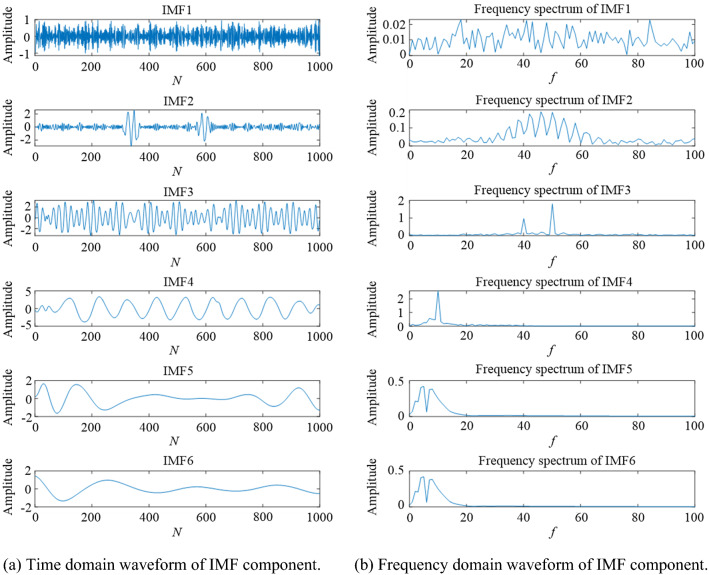
The component waveforms after EMD processing.

In order to further verify the advantages of VMD decomposition based on parameter optimization algorithm over EMD, 5 dB noise signal is selected as an example for specific analysis. Figures 12 and 13 show the waveforms of signal components decomposed by VMD and EMD, respectively, where the parameters of VMD are obtained by using the fused parameter optimization algorithm TS-WOA-VMD. It is evident from Fig. 12(a) that the original noise-containing signal is decomposed into several IMFs, among which IMF1, IMF2, and IMF3 belong to relatively stable sub-sequences with different frequency scales. Based on the frequency domain analysis presented in Fig. 12, it is evident that their corresponding frequencies are exactly the three frequency values of simulation signal *c*_*2*_(*t*), i.e., 10 Hz, 50 Hz, and 40 Hz. For the IMFs obtained by EMD processing, the time-domain and frequency-domain waveforms in Fig. 13 show that there is frequency aliasing of IMF3 component. The serious endpoint effect appears on the left side of IMF4 and the energy leakage can be seen in the frequency domain corresponding to IMF4.These problems affect the decomposition accuracy of EMD to a certain extent. The VMD decomposition based on parameter optimization obtains better modal components adaptively, which effectively improves the ability of signal denoising.

## Application of machine tool vibration test

**Figure 14:**
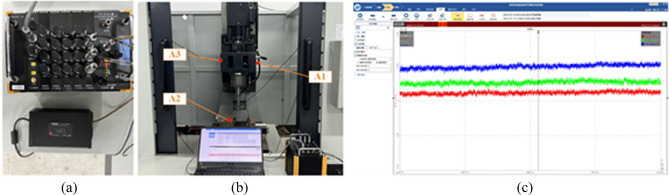
Vibration information acquisition experiment of machine tool.

The actual signal source is based on the vibration data collected by the machine tool multi-source information acquisition platform. The experimental environment is located in a common machine tool workshop. There are multiple machines operating simultaneously and the personnel flow is high as well. There is a large amount of unknown noise in the workshop, which seriously impacts the vibration signals and is not conducive to the analysis of the performance of machine tools. The collected vibration signals are denoised based on the proposed TS-WOA-VMD-CA-WT method. In order to verify the feasibility and superiority of the proposed method, we compare it with other denoising methods. The vibration signal acquisition system used in the experiment is composed of IEPE piezoelectric acceleration sensor, DHDAS dynamic data acquisition and analysis device, and computer. The acquisition and analysis device is presented in Fig. 14(a). The vibration of each part of the machine tool is monitored by using the magnetic suction acceleration sensor and is then connected with the computer through 1394 bus for storage and subsequent analysis and processing. Figure 14(b) shows the sensor measuring point arrangement on the machine tool. The acceleration sensors are placed on the front of the headstock (A1), the side of the headstock (A2), and the bench vice position (A3) of the machine tool for collecting the vibration data for a long time. Figure 14(c) shows the interface of the signal acquisition software used in the experiment.

**Figure 15:**
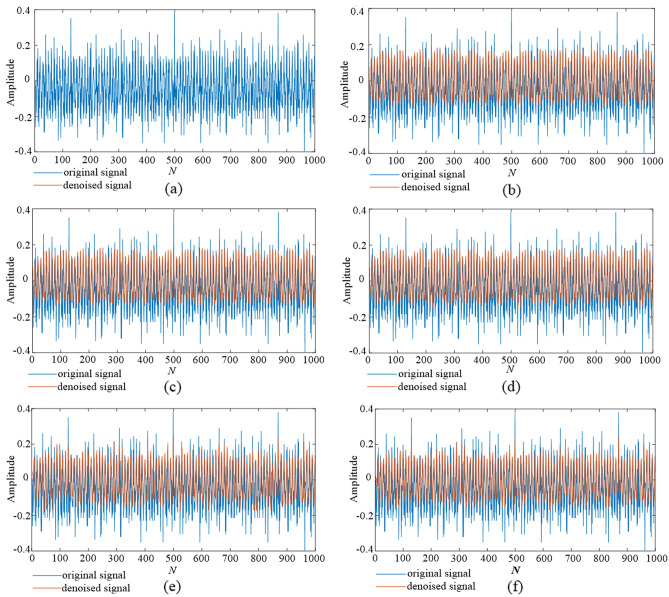
The denoising results of 5 different methods. (**a**) original signal. (**b**) TS-WOA-VMD-CA-WT. (**c**) WOA-VMD-CA-WT. (**d**) TS-VMD-CA-WT. (**e**) EMD-CA-WT. (**f**) EEMD-CA-WT.

The sampling frequency set in the experiment is 1000 Hz. The sampled data with a length of 1000 is randomly selected from three measuring points for denoising.TS-WOA-VMD-CA-WT,WOA-VMD-CA-WT, TS-VMD-CA-WT, EMD-CA-WT, and EEMD-CA-WT methods are used to denoise the three groups of signals. Since the pure vibration signal cannot be obtained, we introduce noise rejection ratio (NRR)^25^ as the evaluation index of signal denoising effect. *NRR* reflects the prominence of effective signals before and after denoising. The larger the value of *NRR*, the better is the denoising effect. The expression of *NRR* is expressed as follows:

14 $$ NRR{ = }10\left(\lg \sigma_{1}^{2} - \lg \sigma_{2}^{2} \right) $$

where, $\sigma_{1}^{2}$ and $\sigma_{2}^{2}$ are the variance of the denoised signal before and after denoising, respectively. In this part the denoised results of a group of signals at the measurement point A1 are selected for visualization, as shown in Fig. 15. The corresponding *NRR* values of the three groups of signals denoised by different methods are shown in Table 4, where group 1, group 2, and group 3 signals are from the measured data of points A1, A2, and A3, respectively.①denotes TS- WOA-VMD-CA.②denotes WOA-VMD-CA. ③ denotes TS-VMD-CA. ④ denotes EMD-CA. ⑤ denotes EEMD-CA. In addition, the table compares the direct signal reconstruction in real vibration signal processing and the secondary processing through wavelet threshold method. ‘ − ’ represents the direct reconstruction, and ‘ + WT’ represents the complete signal denoising method proposed in this paper.

**Table 4:** Evaluation of vibration data of machine tools processed by different denoising methods.

Group		①	②	③	④	⑤
1	−	0.3254	0.3034	0.2528	0.0331	0.2294
	+ WT	4.1755	3.5320	2.9554	2.7097	3.2160
2	−	0.2280	1.0140	0.4035	0.0839	0.0187
	+ WT	2.6870	2.6691	2.5775	2.4208	2.6253
3	−	0.8184	0.6927	0.6731	0.0104	0.0596
	+ WT	0.8184	0.7231	0.7176	0.7924	0.7970

As presented in Fig. 15, the five methods compared in this work have corrected the baseline drift phenomenon in the original signal to a certain extent. In addition, they have also reduced many burrs and protrudes in the original signal. A comparison of these five methods shows that the proposed TS-WOA-VMD-CA-WT retains more useful information in the signal, while reducing the noise. Moreover, the abrupt points in the denoising signal are reduced, and the whole signal becomes relatively smooth and clear. However, the denoising effect of EMD-CA-WT and EEMD-CA-WT is poor, and the noise of abnormal mutation of some signals cannot be removed. Since the intensity of noise added in the three groups of signals is different, the same method will have different NRR values for different signals. However, when processing the same group of data, the TS-WOA-VMD-CA-WT method achieves the optimal effect, and the NRR values of the three groups of denoised signals are 4.1755, 3.4145, and 0.8184, respectively. Table 4 shows that the proposed method has the best denoising effect on the vibration data from different parts of the machine tool. It not only removes the noise signal adaptively, but also has good robustness in terms of adapting the vibration information of various parts of the machine tool. By comparing the signal reconstructed directly with the signal processed by wavelet threshold, we can find that the effect of wavelet threshold processing is better. This is because the result of direct reconstruction of decomposed signals will depend on the set correlation coefficient threshold. Especially for EMD and EEMD methods, direct reconstruction will even lead to almost no denoising effect due to the existence of modal aliasing and white noise residue in their obtained components. When using VMD method, it is also inevitable that the modal components used for reconstruction still carry a large amount of noise. However, the wavelet threshold method can further denoise these components, so as to obtain a better denoising effect. In short, TS-WOA-VMD-CA-WT also has advantages in denoising performance of real signals as compared with other methods.

## Conclusions

In order to reduce the noise mixed in the vibration signals of machine tools, this work proposes a joint analysis denoising method TS-WOA-VMD-CA-WT. The VMD optimized by the new fusion algorithm is used to decompose the signal, and the effective signal components are selected based on the CA and denoised by the three-layer WT. Finally, the denoised signal is obtained by reconstruction. The significance of this study lies in that the useful information collected by the sensor can be extracted as much as possible through a complete set of vibration signal adaptive denoising method, and the discernability of the information can be enhanced. It lays a foundation for subsequent information fusion, and can be used in machine tool characteristic identification and fault diagnosis in the future.

The analysis and comparison of simulation experiment and actual experimental data shows that:

- (1) The proposed denoising method can play a role in the preprocessing of machine tool vibration signals and achieve satisfactory noise suppression effect.
- (2) As compared with the traditional EMD method and the improved EMD method, the VMD can adaptively adjust the optimal mode number according to the application situation, so as to obtain the appropriate mode component.
- (3) As the key parameters of VMD, the penalty factor *α* and the number of decomposition layers *k* have a significant effect on the decomposition. Based on the parameter optimization algorithm proposed in this work, appropriate relevant parameter values can be obtained to improve the subsequent denoising ability.
- (4) As compared with the typical WOA and TS algorithm, the parameter optimization based on fusion algorithm has higher accuracy and stability.
- (5) TS-WOA-VMD-CA-WT, WOA-VMD-CA-WT, TS-VMD-CA-WT, EMD-CA-WT, and EEMD-CA-WT are used to denoise different signals mixed with different noise intensity. In addition, it is verified that the proposed method have larger SNR and smaller root mean square error. Therefore, its denoising ability is stronger.

## Data Availability

The data that support the findings of this study are openly available at https://github.com/chenyushen12138/denoising.git.
