# Author Correction: Denoising method of machine tool vibration signal based on variational mode decomposition and Whale-Tabu optimization algorithm

**DOI:** 10.1038/s41598-023-29353-x

**Published:** 2023-02-06

**Authors:** Chengzhi Fang, Yushen Chen, Xiaolei Deng, Xiaoliang Lin, Yue Han, Junjian Zheng

**Affiliations:** 1 Key Laboratory of Air-Driven Equipment Technology of Zhejiang Province, Quzhou University, Quzhou, 324000 China; 2 College of Mechanical Engineering, Zhejiang University of Technology, Hangzhou, 310023 China

Correction to: *Scientific Reports* 10.1038/s41598-023-28404-7, published online 27 January 2023

The original version of this Article contained an error in the Method section,

“The position update method is judged according to the value of |A|, the update method referring to (8), (9), (10), and (11).”

now reads:

“The position update method is judged according to the value of |A|, the update method referring to (6), (7), and (8).”

Additionally, in the Simulations,

“The proposed method is compared with other denoising methods, including WOA-VMD-CA-WT, TS-VMD-CA-WT, and EMD-CA-WT.”

now reads:

“The proposed method is compared with other denoising methods, including WOA-VMD-CA-WT, TS-VMD-CA-WT, EMD-CA-WT and EEMD-CA-WT.”

The original Article has been corrected.

